# Sub-chilling methods for Atlantic salmon with 7 days in refrigerated seawater and subsequent sub-chilled storage

**DOI:** 10.1038/s41598-025-09371-7

**Published:** 2025-07-08

**Authors:** Sherry Stephanie Chan, Bjørn Tore Rotabakk, Trond Løvdal, Izumi Sone, Bjørn Roth

**Affiliations:** Department of Processing Technology, Nofima AS, Stavanger, Norway

**Keywords:** Microbiology, Ocean sciences

## Abstract

This study evaluated the sub-chilling of whole gutted Atlantic salmon in refrigerated seawater (RSW) maintained at -1 °C for 7 days, compared to traditional ice storage at 0 °C and RSW storage at -1 °C for 4 days followed by 3 days without ice. After filleting, portioning, and modified atmosphere packaging, the initial RSW-stored fish were continuously stored at -1 °C, while the initial ice-stored fish were stored at refrigerated temperatures at 4 °C. The 7-day RSW storage resulted in a significant weight gain, higher water-holding capacity (WHC), and increased water and salt content. Ice-stored fish had higher calpain activity. After packaging, drip loss was highest for the 4-day RSW-stored fish. The 7-day RSW-stored fish demonstrated consistently better WHC and water content. The CO_2_ levels decreased, while O_2_ levels increased more rapidly within the package for the RSW-stored fish. There was also a significant extension in the microbiological and sensory shelf life for the RSW-stored fish. *Photobacterium* was the dominant bacterium in all storage methods. This study highlights the potential to extend salmon shelf life with sub-chilling, significantly reducing ice usage.

## Introduction

The Norwegian aquaculture industry has rapidly increased from a production of 4000 tonnes since the 1970 s to around 1.5 million tonnes in 2022^1^. Therefore, maintaining good fish quality is already essential from the beginning of the value chain. Sub-chilling is a food preservation method that lowers the product’s internal temperature to < 0 °C and can be applied to whole fish and fillets. Researchers also refer to sub-chilling as super-chilling, which is often defined as lowering the temperature below the product’s freezing point, causing partial ice crystallization^2–4^.

Refrigerated seawater (RSW) systems are practical for sub-chilling whole fish onboard fishing vessels, effectively chilling large volumes of species, like salmon, mackerel, herring, tuna, halibut, and shellfish^5,6^. Studies on the quality of chilling various fish species in RSW were published between the 1960 s and 1990s^7–11^. Previous research has also discussed the beneficial effects of RSW storage compared to ice, as ice can lead to unwanted attributes like flesh bruising and leaching of nutrients^5,12,13^. Recent studies have extensively examined the industrial practice of sub-chilling Atlantic salmon and Atlantic cod to −1 °C in RSW systems onboard fishing vessels^14–17^. These studies showed the possibility of extending the fish’s shelf life through RSW storage while maintaining freshness. Nevertheless, good circulation and hygiene within the tanks are important to obtain uniform chilling profiles and prevent contamination.

The freezing point of seawater with 3.5% salinity is around − 2 °C. Therefore, lowering the RSW temperature to sub-chilled conditions at −1 °C prevents ice crystal formation. In addition, seawater surrounds each fish and gives an effective cooling and washing effect that can be beneficial for further processing^5^. The pumpability to and from the RSW systems requires minimal and more hygienic fish handling^5,13^. However, salt uptake in RSW storage can be a limiting factor. This uptake depends on several factors, such as species, size, gutted or non-gutted, storage time, and loading density. Gokoglu and Yerlikaya^5^ reported an upper limit of salt uptake of 0.5% in raw fish. In addition, the large size and subcutaneous fat layer of fatty fish like salmon hinder salt migration^18^.

Previous studies on RSW storage of Atlantic salmon simulated a realistic sea transport for up to 4 days^16^. However, the impact of extending the RSW storage beyond this period, combined with sub-chilled storage after processing and packaging, has not been studied. This study aimed to examine the storage of whole gutted salmon in RSW for 7 days at −1 °C, followed by modified atmosphere packaging (MAP) after filleting and storage in sub-chilled conditions at −1 °C (group S). This process was compared against two groups: traditional storage on ice for 7 days at 0 °C, then at 4 °C after MAP (group I), and RSW sub-chilling for 4 days, then 3 days without ice at −1 °C, and sub-chilled at −1 °C after MAP (group R). Quality parameters periodically analyzed include gas composition, drip loss (DL), water holding capacity (WHC), water and salt content, colour, enzyme activity, sensory analysis and microbiological diversity, quality, and shelf life.

## Materials and methods

### Raw material and processing

**Fig. 1:**
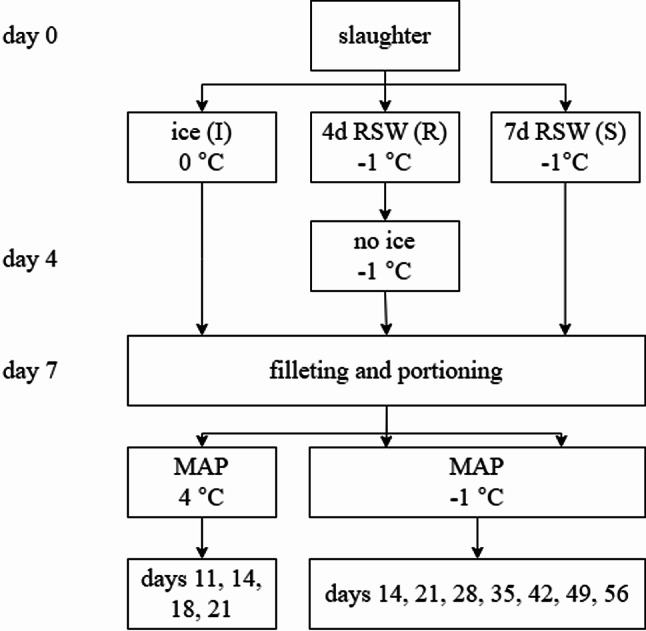
A graphical illustration of the experimental timeline. RSW and ice represent storage in refrigerated seawater and ice, respectively. MAP represents modified atmosphere packaging. Groups I, R and S represent storage in ice, 4 days in RSW before packaging in EPS boxes without ice, and 7 days in RSW, respectively.

Part of the results from groups I and R were published in the study of Chan, et al.^17^. The present study was conducted in parallel as a supplementary study for group S. Before the experiment, RSW was made in the laboratory in a 1000 L polyethylene tank containing self-made brine (3.5% salinity). 88 whole gutted Atlantic salmon were obtained from a local slaughter facility (starved for 14 days, average weight: 4.51 ± 0.3 kg). The right fillets of 5 fish were used for sampling on day 0 (Fig. 1). In contrast, 4 of the left fillets were used to determine the freezing point of the raw material by placing temperature loggers (Testo 176-T4, Max Sievert A/S, Norway) into the fillets and storing them at −30 °C for 1 day. The remaining fish were split into 3 groups, where 45 fish were kept in RSW for 4 days (group R) at −1 °C before being stored in expanded polystyrene (EPS) boxes without ice for 3 more days. Next, 8 fish were kept in RSW for 7 days (group S). The control group was fish in EPS boxes containing ice (*n* = 30) kept at 0 °C. Each group recorded individual weights on random fish to follow the weight development on day 4 and day 7. Temperature loggers (TrackSense Pro, Ellab A/S, Denmark) were inserted randomly from each group.

**Fig. 2:**
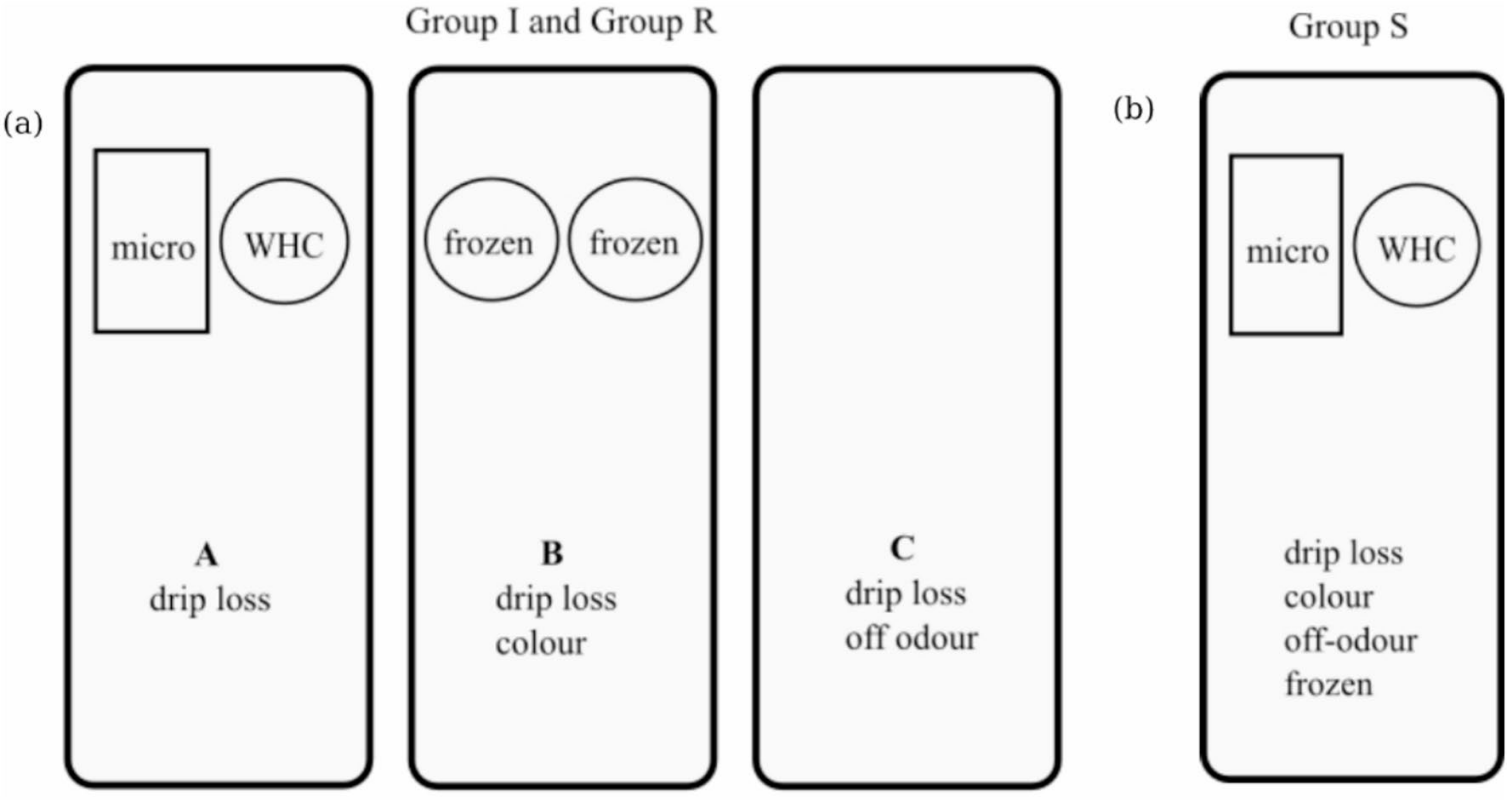
(**a**) A graphical illustration of quality analyses done on fillet portions A, B and C coming from the same right fillet of groups I and R. This figure is adapted from Chan, et al.^17^. (**b**) A graphical illustration of portions obtained from the right and left fillets for group S. “Micro” and “WHC” represent microbiology and water holding capacity analyses. “Frozen” indicates samples frozen at −80 °C for further analysis of salt content and enzyme activity.

On day 7, all fish were manually filleted and portioned. The right fillets were used for groups I and R, portioned into 3 portions per fillet (portions A, B, and C, *n* = 60 portions for group I, *n* = 105 portions for group R). For group S, both right and left fillets were used. These fillets were also portioned into 3 to 4 portions per fillet and then randomized (*n* = 37 portions). All portions were then packaged in modified atmosphere packaging (MAP) with 60% CO_2_:40% N_2_ gas mixture, with a gas-to-product (g/p) ratio of 2, in crystallized polyethylene terephthalate (CPET) trays (C2187-F black, 680 ml, Færch, Denmark). Group I was stored at 4 °C, while groups R and S were stored in sub-chilled conditions at −1 °C. A Multivac T200 Tray sealer (Multivac, Germany) sealed the trays using a PET sealant (Cryovac OSF33ZA, thickness 33 μm, oxygen permeability 60 cm^3^/m^2^/24 h/bar (23 °C, 0% RH), Sealed Air, Norway). For group I, quality analysis was done periodically on days 11, 14, 18, and 21. For groups R and S, quality analysis was done weekly on days 14, 21, 28, 35, 42, 49, and 56. Figure 2a and b illustrate the quality analyses carried out during the sampling days on groups I, R, and S, respectively.

### Physical and sensory analyses

#### Headspace gas, drip loss, and water holding capacity

For each sampling day, portions A, B, and C were chosen from the same fish (*n* = 5/group) for groups I and R. In contrast, 5–6 random portions were chosen for group S. Headspace gas was measured before opening the packaging using a PBI Dansensor CheckMate 9900 Headspace gas Analyzer (Nordic Supply System, Norway). Drip loss (DL) was measured on all portions immediately after opening the packages, calculated by the % weight change from its initial weight. Water holding capacity (WHC) was sampled based on the method by Skipnes, et al.^19^ where muscle samples were stamped with a metal cylinder (diameter 31 mm) and transversally sliced into 2 pieces. The top piece underwent a low-speed centrifugation process at 1800 rpm, 15 min, 4 °C to determine the weight loss after centrifugation. The bottom piece underwent water content analysis by drying the sample at 105 °C for 18 h.

#### Salt content

The salt content was determined from the frozen samples on days 0, 4, 7, and 49 with an EasyCl titration system (Mettler Toledo, Norway). The titration agent used was 0.1 M AgNO_3_. 100 mL of warm deionized water was added to around 1.5 g of sample. The mixture was then homogenized for 40 s at 13,500 rpm using an Ultra Turrax T25 (Janke & Kunkel IKA, Labortechnik, Staufen, Germany). 1 M HNO_3_ was added to the resulting mixture, and automatic titration was done. Titration stops until the equivalence point is reached, where AgCl is formed. The % salt is calculated based on the formula: (Eq-B)*T*M*F1/W, where Eq is the volume (ml) of AgNO_3_ used at equivalence point; B = 0, blank value; T = 0.1 mol/l, the concentration of titrant; M = 58.44 g/mol, the molecular weight of NaCl; F1 = 0.1, the conversion factor for % and W = sample weight (g).

#### Colour

Colour was analyzed using a digital imaging system, DigiEye (VeriVide Ltd, UK), connected to a Nikon D80 digital camera (35 mm lens, Nikon Corp., Japan). Samples were analysed in a lightbox connected to a 6400 K light. Digipix software incorporated in the system was used to measure the L*a*b* values from the images, where L*, a*, and b* represent lightness, redness, and yellowness, respectively^20^.

#### Sensory analysis

Sensory analysis was performed by a semi-trained internal panel (*n* = 4 to 5) on every sampling day after packaging. Samples were re-packaged in plastic pouches (PA/PE, 160 × 200 mm, LietPack, Lithuania) and randomly marked with a three-digit code. The evaluation was based on off-odour analysis obtained from the “smell” attribute of the fillet index method, where demerit points are given for key attributes “smell, gaping, colour, consistency, and surface”. The samples were ranked on a scale of 0 to 3, where 0 and 3 indicate a fresh and spoiled product, respectively.

### Enzyme analysis

Crude enzyme extract was obtained from three samples of each treatment group from days 0, 4, and 7, as described in Yang, et al.^21^ with slight modifications. Approximately 5 g of each sample was homogenized in 20 mL Elix water using a T25 digital Ultra Turrax equipped with the S25 N 8G ST probe (IKA, Staufen, Germany) at 22,000 rpm for 20 s. The homogenate was then placed at 4 °C for up to 30 min with stirring at 10 min intervals before centrifugation at 20,000 × g for 20 min at 4 °C (Heraeus Multifuge x3 FR, ThermoFisher Scientific). The protein content of the resulting supernatant as a crude enzyme extract was determined using the Lowry protein assay (Modified Lowry Protein Assay Kit, ThermoFisher Scientific). The crude extracts were stored at −80 °C until analysis. Sample supernatants were thawed and centrifuged at 17,000 × g for 60 s (Heraeus Fresco 17 Microcentrifuge, ThermoFisher Scientific) before use.

Enzyme activities of cathepsin B/L, calpain, and collagenase were measured according to Yang, et al.^21–23^ with modifications. Fluorogenic substrates N-Suc-Leu-Tyr-AMC for calpains, Z-Phe-Arg-AMC for cathepsin B/L, Suc-Gly-Pro-Leu-Gly-Pro-AMC for collagenases (Bachem Holding AG, Bubendorf) were used. 100 µL of the extract was mixed with 100 µL of respective assay buffer, followed by further incubation at 30 °C for 10 min before 100 µL of 90 µM substrate (dissolved in Elix water) was added to activate the reaction at 30 °C for 15 min. The assay buffer for cathepsin B/L was 150 mM Bis-Tris, 30 mM EDTA, 6 mM dithiothreitol, and pH 6.0. The collagenase and calpain assay buffer consisted of 150 mM bis-Tris, 7.5 mM CaCl_2_, and pH 6.0. The reaction was terminated by transferring 1 mL stop buffer (1 mM monochloroacetic acid, 30 mM acetic acid, and 70 mM sodium acetate), and the extract solution was placed on ice for 10 min. The standard curve was constructed in the appropriate concentration range using 7-amino-4-methyl coumarin (AMC) (Bachem Holding AG, Bubendorf) in stop buffer. A sample blank was prepared by replacing the sample extract with an equal part of Elix water mixed with assay buffer, substrate, and stop buffer in the respective order. The fluorescence of AMC was measured at 460 nm after excitation at 380 nm using the Synergy H1 Multi-Mode Microplate Reader (BioTek, VT, USA). Enzyme activities were expressed in the sample as µM (for cathepsin B/L and collagenase) or nM (for calpain) AMC/mg protein.

### Microbiological analysis

Microbiological analysis was done on all groups using the NMKL method No. 184^24^ to quantify for total psychrotrophic (TPC), total mesophilic (TMC), and hydrogen sulphide producing bacteria (HSPB). Around 10 g of sample was collected, diluted 10x using sterile peptone water, and then homogenized in a Smasher^®^ (AES Laboratorie, bioMérieux Industry, St. Louis, MO, USA) for 2 min. Appropriate dilution series were made, and 49.2 µl was transferred to Long and Hammer (L&H) agar using an automatic Eddy Jet 2 W Spiral Plater (IUL micro, Barcelona, Spain) to quantify for TPC. 1 mL was transferred to iron agar containing 0.04% L-cysteine (Sigma Aldrich, Oslo, Norway) to quantify for TMC and HSPB. The L&H agars were incubated at 15 °C for 7 days, while iron agars were at 25 °C for 72 h. Microbial concentrations are presented in log cfu/g.

### DNA extraction and illumina partial 16 S rRNA gene sequencing

45 mL of the homogenates remaining from the microbiological analysis from samples from days 14, 21, and 49 were transferred to sterile 50 mL Falcon tubes. The tubes were filtered through a Whatman 589/1 filter paper (Whatman, Maidstone, UK) and centrifuged (Heraeus Multifuge X3 FR, VWR International AS, Oslo, Norway) with a fixed angle rotor (Fiberlite F15-8 × 50cy Rotor, Thermo Scientific, Oslo, Norway) at 261 × g for 5 min at 4 °C. The supernatant was transferred to a new Falcon tube and centrifuged at 6534 × g for 20 min at 4 °C, and the supernatant was discarded. The remaining pellet was suspended in 1.5 mL of deionized water. The final suspension was centrifuged at 12 225 × g for 2 min (Eppendorf MiniSpin, Eppendorf Norway AS, Oslo, Norway). The supernatant was discarded, and the final pellets were kept at −80 °C for further analysis. The protocol for microbiota analysis was carried out according to Chan, et al.^17^. Briefly, the pellets were homogenized by tissue homogenizer (Precellys Evolution 24 Tissue homogenizer) for 3 × 40 s at 7400 × g with 10 s between intervals. DNA extraction was done with the DNeasy PowerLyser PowerSoil kit (Qiagen, Hilden, Germany). 16 S rRNA gene PCR (V4 region) and paired-end sequencing (2 × 150 bp) was performed using the protocol published by Caporaso, et al.^25^ and the sequences were processed in QIIME2^26^. To obtain an estimate of the microbial development of the dominating bacterial species (taxa above 0.02%) during storage, the log of the relative abundance values multiplied with the total aerobic counts (log_10_ (relative abundance × CFU/g), was calculated.

### Statistical analysis

A general linear model (GLM) was used for statistical analysis in Minitab^®^ version 21.1.1 (Minitab, USA). The groups (I, R, S) were set as categorical factors and storage days as independent variables. A one-way analysis of variance (ANOVA) with post hoc Tukey’s pairwise comparison test was carried out for WHC and water content results on day 7. For the sensory analysis, judges were included as an additional categorical factor. A t-test was used to compare salt contents on day 49. In addition, Pearson’s correlation (r) was used between selected variables. The significance of the difference between means in microbiota analysis was analysed by t-test in Microsoft Excel. All results are presented as mean ± standard deviation, and the p-value was set to 0.05.

## Results and discussion

**Fig. 3:**
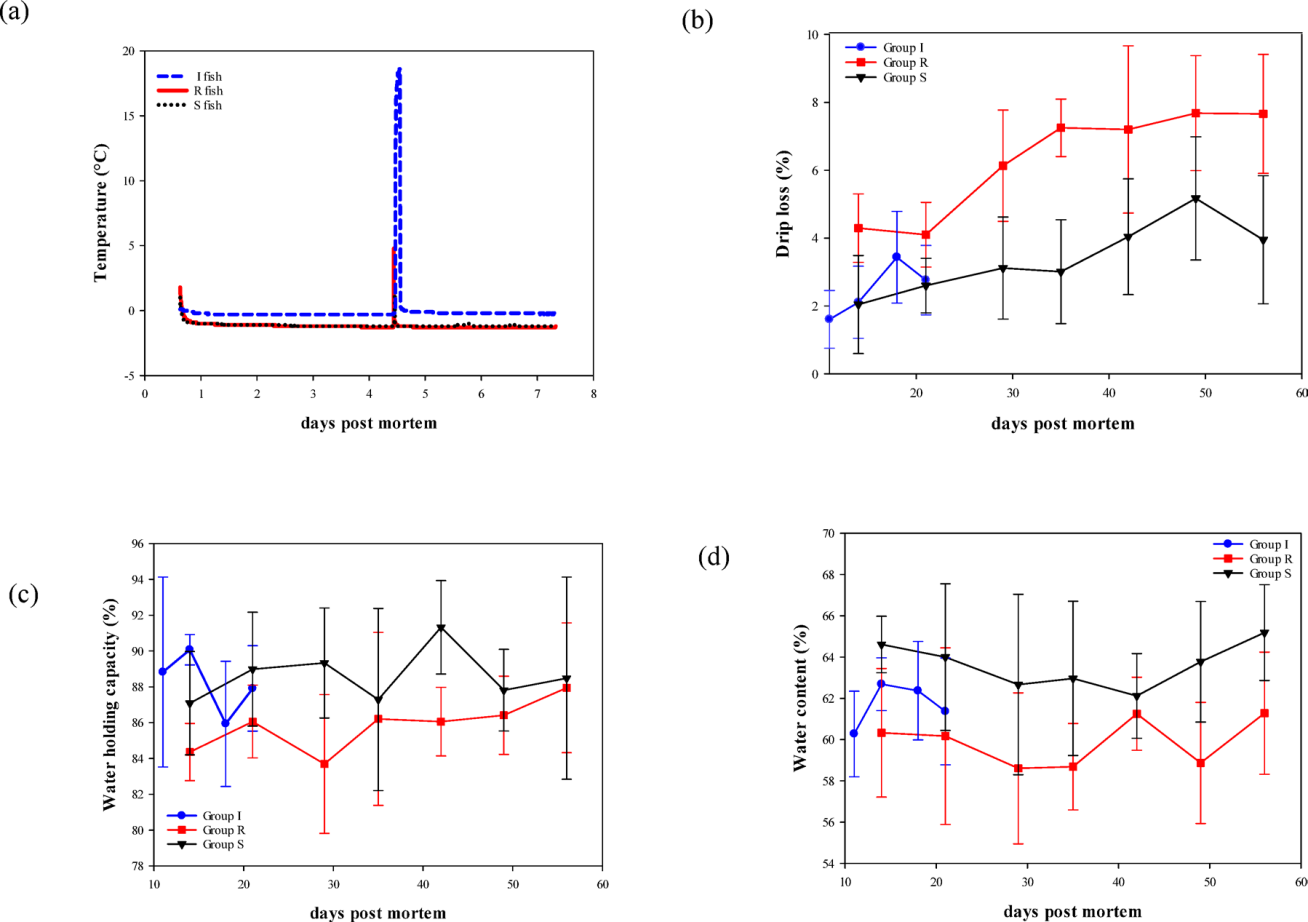
Graphs showing (**a**) temperature development of whole fish for 7 days of storage. (**b**) Drip loss (%) of portions after packaging (GLM; days: *p* < 0.001, group: *p* < 0.001). (**c**) Water holding capacity (%) of portions after packaging (GLM; days: *p* = 0.620, group: *p* = 0.001). (**d**) Water content (%) of portions of packaging (GLM; days: *p* = 0.365, group: *p* < 0.001).

The freezing point of salmon was measured at −1.3 ± 0.2 °C (*n* = 4). The temperature profiles for groups R and S were constant at −1 °C for 7 days, while the iced fish remained constant at 0 °C (Fig. 3a). The observed temperature spike on day 4 was due to the removal of fish for weighing and sampling. Nevertheless, the sub-chilled fish maintained a temperature of −1 °C during sub-chilled storage despite the sub-chilled group being removed from the RSW on day 4.

### Water holding properties and salt content

Table 1 shows the results summary for the different whole fish storage until day 7 before filleting. There was a significant difference in weight changes (*p* < 0.001) and storage days (*p* < 0.001), and weight increased by 3.0 ± 0.4% for salmon stored in RSW for 7 days. In contrast, the control group on ice has a relatively stable weight loss of 0.3 ± 0.2% and 0.4 ± 0.3% on days 4 and 7, respectively. Salmon stored in RSW for 4 days had a weight gain of 1.7 ± 0.3% before losing weight when stored in EPS boxes for another 3 days, resulting in an overall weight gain of 0.4 ± 0.3%. A previous study on pink salmon stored in RSW for 10 days at −0.6 °C showed an overall weight gain of 6% ^10^. In addition, Erikson, et al.^27^ studied the weight changes of gutted salmon at −2 °C in a seawater slurry for 11 days and found a similar weight increase of 2.5 and 6% on days 4 and 11, respectively. However, when these fish were stored in the air for 3 h after 11 days, the mean weight gain dropped to 2.4%. Similar to the findings of our study, this indicated that some water is lost by removing the fish from RSW storage, possibly due to more uptake of free water that can be loosely bound in the muscle and easily lost.

WHC generally decreased (*p* < 0.001), while there were no significant differences in water content (*p* = 0.103) through whole fish storage. On day 7, groups R and S had a significantly higher WHC (*p* < 0.001) and water content (*p* = 0.016) than the iced-stored fish. However, continuously storing fish in RSW for 7 days gave a similar WHC as those stored for 4 days. In line with previous studies, WHC increased after 4d-RSW storage and was most prominent on day 7 due to the salting in the process, as RSW can be considered a brine with low salt concentration^28,29^.

The initial salt content of fish on day 0 was at 0.2 ± 0.0%. As fish were kept in RSW for 4 and 7 days, the salt content increased by 0.1% when measured on day 7 (*p* = 0.005). After packaging, the salt contents on day 49 for group R remained at 0.3 ± 0.0%, while group S continued to increase to 0.6 ± 0.1% (*p* < 0.001). Salt uptake was expected since fish were in direct contact with the seawater, resulting in a concentration difference and salt penetrating through the fish skin and exposed belly cavity by osmosis. Since the exposure to salt water was longer for group S, the salt content was double that stored for 4 days in RSW.

Figure 3b shows the drip loss of fillet portions after modified atmosphere packaging. There was a significant increase in drip loss through storage (*p* < 0.001). Measuring drip loss is time-dependent, and storage time should be minimized^30^. Group R was observed to have higher drip loss (*p* < 0.001) and lower WHC (*p* = 0.001) and water content (*p* < 0.001) than groups I and S through chilled and sub-chilled storage (Fig. 3b and d). Storage days did not influence WHC (*p* = 0.620) or water content (*p* = 0.365). In addition, the salt content was positively correlated with water content (*r* = 0.730) and negatively with drip loss (*r*=−0.702). This suggests that storing fish in RSW for 7 days seemed to retain water better after processing, possibly explained by the higher salt absorbed into the muscle.

**Table 1:** Weight gain (%), water holding capacity (%), water content (%) and colour results for groups I, R and S on days 0, 4 and 7 post mortem.

	Group I			Group R			Group S			Effect^a^
Day	0	4	7	0	4	7	0	4	7	
**Weight gain (%)**	-	−0.3 ± 0.2	−0.4 ± 0.3	-	1.7 ± 0.3	0.4 ± 0.3	-	1.7 ± 0.3	3.0 ± 0.4	*p* < 0.001*
**Effect** ^**b**^	*p* < 0.001*									
**WHC (%)**	93.6 ± 0.8	92.0 ± 1.1	85.4 ± 1.7^A^	93.6 ± 0.8	90.8 ± 2.0	91.6 ± 2.3^B^	93.6 ± 0.8	90.8 ± 2.0	91.3 ± 1.2^B^	*p* < 0.001*
**Effect** ^**b**^	*p* = 0.030*									
**Water content (%)**	61.7 ± 1.3	60.3 ± 2.4	57.4 ± 2.6 ^A^	61.7 ± 1.3	61.3 ± 2.6	62.4 ± 1.4 ^AB^	61.7 ± 1.3	61.3 ± 2.6	60.2 ± 2.8 ^B^	*p* = 0.103
**Effect** ^**b**^	*p* = 0.087									
**Salt content (%)**	0.2 ± 0.0	0.2 ± 0.0	0.2 ± 0.1	0.2 ± 0.0	0.2 ± 0.1	0.3 ± 0.1	0.2 ± 0.0	0.2 ± 0.1	0.3 ± 0.1	*p* = 0.005*
**Effect** ^**b**^	*p* = 0.045*									
Lightness (L*)	66.6 ± 1.1	61.9 ± 2.1	67.1 ± 2.1	66.6 ± 1.1	62.3 ± 1.4	66.0 ± 0.7	66.6 ± 1.1	62.3 ± 1.4	66.2 ± 1.0	*p* < 0.001*
**Effect** ^**b**^	*p* = 0.707									
Redness (a*)	30.2 ± 2.0	33.0 ± 1.1	34.0 ± 2.9	30.2 ± 2.0	33.1 ± 2.4	34.9 ± 1.3	30.2 ± 2.0	33.1 ± 2.4	34.4 ± 1.1	*p* = 0.001*
**Effect** ^**b**^	*p* = 0.741									
Yellowness (b*)	28.1 ± 1.8	29.5 ± 1.2	31.3 ± 2.5	28.1 ± 1.8	29.7 ± 2.7	31.0 ± 0.8	28.1 ± 1.8	29.7 ± 2.7	30.4 ± 0.8	*p* = 0.018*
**Effect** ^**b**^	*p* = 0.368									

^a^significant difference among storage days.

^b^significant difference among groups.

^A, B^significant difference among groups using one-way ANOVA and Tukey’s pairwise comparison test.

*denotes a significant difference with a p-value set to 0.05

### Gas composition

**Fig. 4:**
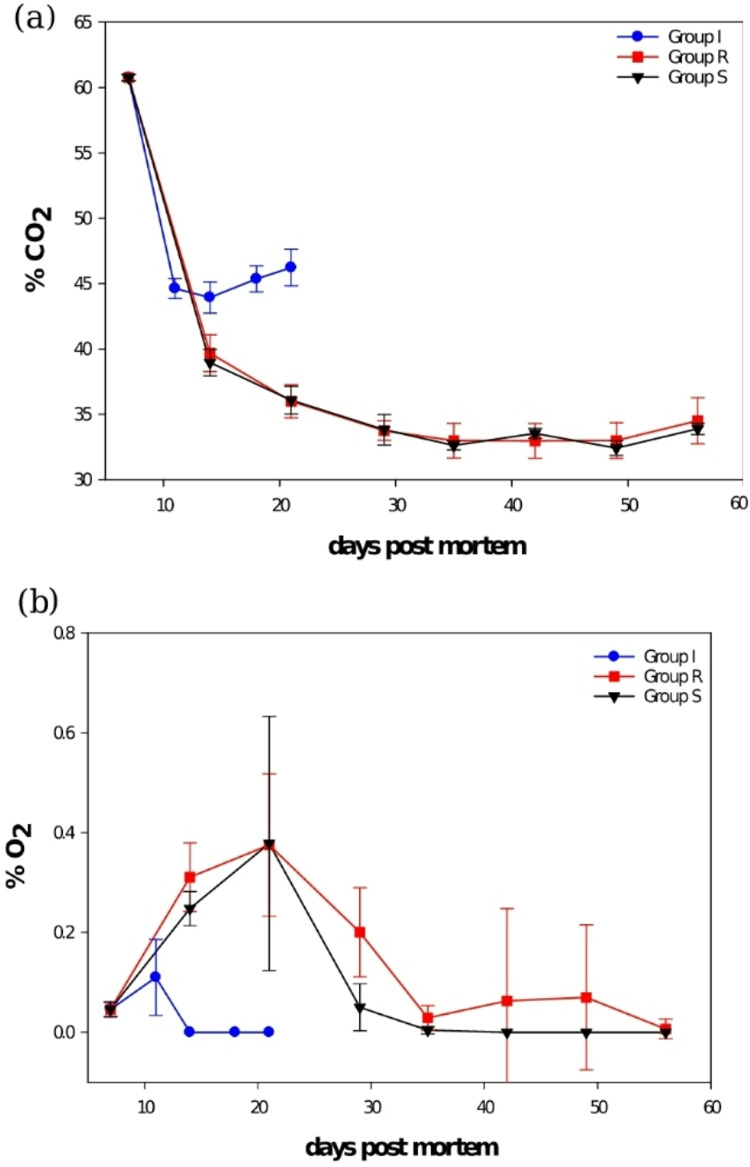
Graphs showing (**a**) % CO_2_ in headspace gas after packaging (GLM; days: *p* < 0.001, group: *p* < 0.001). (**b**) % O_2_ in headspace gas after packaging (GLM; days: *p* < 0.001, group: *p* < 0.001).

Immediately after packaging on day 7, the gas composition contains 60.8 ± 0.3% CO_2_ and 39.2 ± 0.2% N_2_ (residual O_2_ 0.1 ± 0.0%). After 14 days of storage, %CO_2_ (Fig. 4a) and %O_2_ (Fig. 4b) in the headspace decreased to 43.9 ± 1.2% and 0.0% for the control group. This trend was similar for groups R and S after 49 days of storage, with 33.0 ± 1.4% and 32.4 ± 0.6% CO_2_, respectively (days: *p* < 0.001, group: *p* < 001). As the packages were stored for longer, %CO_2_ slowly increased to around 35% on day 56. This can be explained by the aerobic respiration of microorganisms in the product, thereby absorbing the available O_2_. The amount of CO_2_ was higher, while O_2_ was lower for the control group than groups R and S (days: *p* < 0.001, group: *p* < 001). This is related to the different storage temperatures, as the control group was stored in chilled conditions at 4 °C. In contrast, groups R and S were stored in sub-chilled conditions at −1 °C. This observation was similar to Sivertsvik, et al.^31^ and is due to the solubility of CO_2_ in the water phase of the muscle increasing with decreasing temperature^32–34^. CO_2_ has antimicrobial and antifungal properties^31^. Its application inhibits food spoilage microorganisms^33^ especially with high-barrier films. This suggests that the increase in CO_2_ solubility at lower storage temperatures improves the effectiveness of MAP.

### Colour

No significant differences in colour were observed among the groups from days 0 to 7 during whole fish storage (Table 1). The lightness of whole fish decreased from day 0 to day 4 before increasing to day 7 (*p* < 0.001). A similar observation was seen in Chan, et al.^28^as explained by the influence of reflective properties due to loss of fillet translucency after slaughter.

From the data provided in Table 2, group R was slightly lighter than group S after portioning and packaging, and group I gave the lightest (*p* < 0.001) and least yellowish colour (*p* = 0.006) on days 14 and 21. There was a significant decrease in yellowness through storage days (*p* = 0.034), in line with the results from previous studies^28,35^.

**Table 2:** Lightness (L*), redness (a*) and yellowness (b*) for groups I, R and S after portioning and packaging from day 11 post mortem.

day	Group I			Group R			Group S			Effect^a^		
	L	a	b	L	a	b	L	a	b	L	a	b
11	65.3 ± 1.3	32.8 ± 1.0	30.0 ± 0.8	-	-	-	-	-	-	< 0.001*	0.649	0.034*
14	65.8 ± 1.8	31.8 ± 1.1	28.5 ± 0.7	65.5 ± 1.3	32.5 ± 1.0	29.4 ± 0.6	62.5 ± 1.0	33.4 ± 1.7	30.4 ± 1.2			
18	66.2 ± 1.7	31.7 ± 2.2	27.9 ± 1.6	-	-	-	-	-	-			
21	63.9 ± 2.4	30.6 ± 2.9	27.3 ± 1.9	62.7 ± 2.2	30.8 ± 1.2	27.9 ± 0.8	59.5 ± 2.2	33.8 ± 1.2	30.8 ± 1.3			
29	-	-	-	68.4 ± 1.6	32.4 ± 1.5	28.2 ± 0.9	64.9 ± 1.1	32.1 ± 1.5	30.3 ± 1.7			
35	-	-	-	66.3 ± 1.2	35.0 ± 3.6	31.4 ± 3.3	65.1 ± 1.5	31.8 ± 2.3	30.1 ± 2.1			
42	-	-	-	65.8 ± 0.6	34.0 ± 1.1	29.7 ± 1.4	66.7 ± 0.7	32.4 ± 1.2	29.2 ± 1.4			
49	-	-	-	66.1 ± 1.0	32.3 ± 1.4	29.5 ± 0.8	64.3 ± 1.8	33.2 ± 1.1	30.2 ± 1.8			
56	-	-	-	64.9 ± 1.7	33.3 ± 3.0	28.2 ± 3.1	61.4 ± 2.0	31.6 ± 1.4	28.2 ± 1.0			
**Effect** ^**b**^	< 0.001*	0.130	0.006*									

^a^significant difference among storage days.

^b^significant difference among groups.

*denotes a significant difference with a p-value set to 0.05.

### Enzyme analysis

**Fig. 5:**
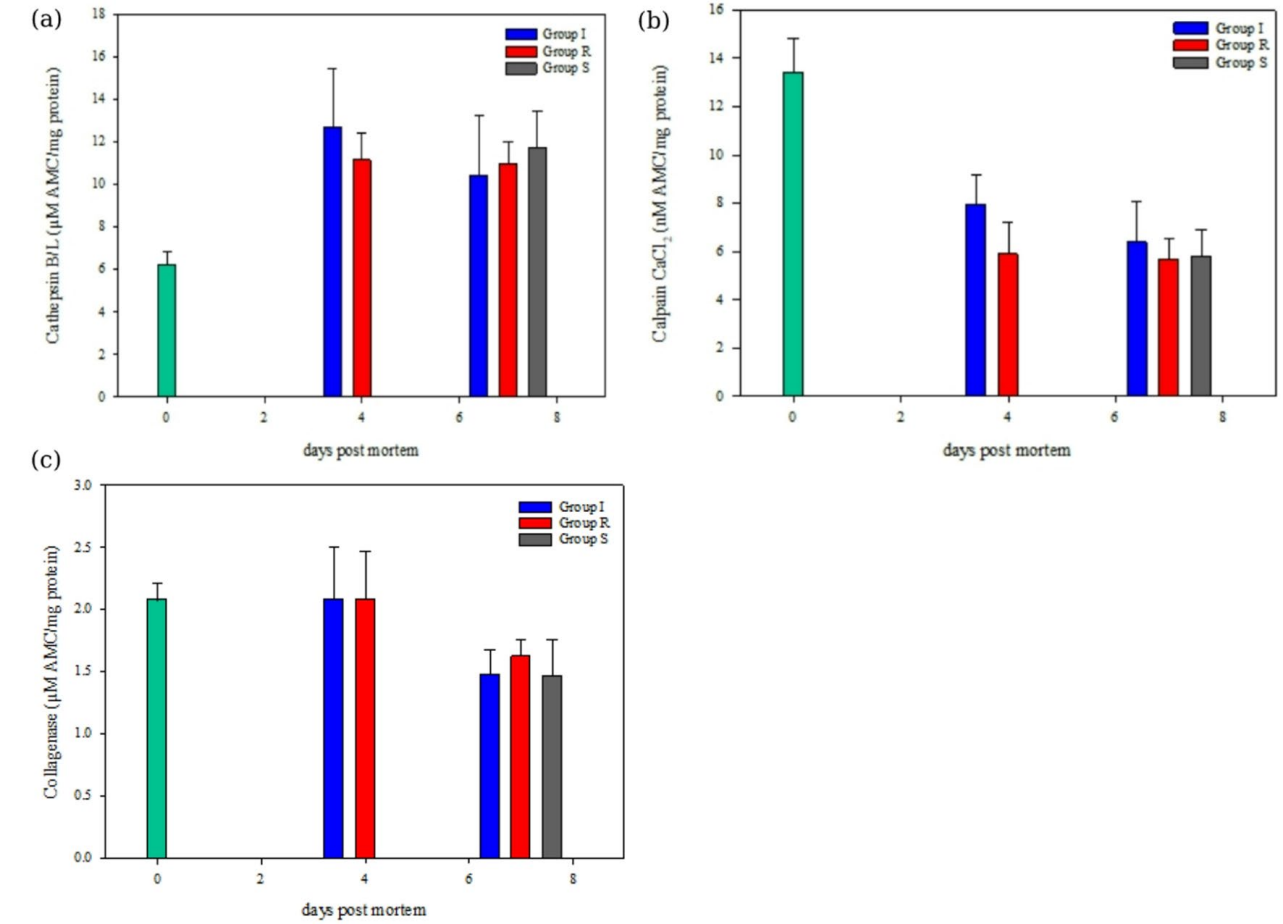
Graphs showing enzyme activity for groups I, R and S on days 0, 4 and 7 post-mortem. (**a**) Cathepsin B/L (GLM; days: *p* < 0.001, group: *p* = 0.521) (**b**) Calpain CaCl_2_ (GLM; days: *p* < 0.001, group: *p* < 0.001) and (**c**) Collagenase (GLM; days: *p* < 0.001, group: *p* = 0.506).

Cathepsin B/L activity showed a significant increase (*p* < 0.001) from 6.2 ± 0.6 µM AMC/mg protein on day 0 to 11.1–12.6 µM AMC/mg protein on day 4 across all groups (Fig. 5a). By day 7, the activity stabilized around 10.4–11.7 µM AMC/mg protein. These results indicate that most protease releases occurred within the first 4 days of storage, regardless of treatment group. Lysosomal proteases play a key role in postmortem muscle degradation and softening. Prolonged storage leads to rupture of lysosomal membranes and release of proteolytic enzymes, exacerbated by postmortem pH change, formation of disruptive ice crystals, and myocyte apoptosis^36–39^. Besides the well-established storage effect, the differences in treatment (*p* = 0.521) did not account for the significantly higher drip loss or lower WHC in the R group during the subsequent storage. Similarly, the sub-zero temperature in RSW did not delay cathepsin activity compared to ice storage, contrary to findings by Chan, et al.^28^. The authors associated the delayed cathepsin activity with the lower refrigerated temperature of RSW. Furthermore, transferring the R group to air storage did not enhance cathepsin activity during the initial 7-day storage or at subsequent storage. Previous studies suggest that changing chilling medium from liquid (as in RSW) to air has been associated with softening and accelerated bacterial growth^27,40^.

Unlike cathepsin activity, calpain (*p* < 0.001) and collagenase (*p* < 0.001) activities generally decreased over the 7-day storage period. Calpain activity declined from 13.4 ± 1.4 µM AMC/mg protein on day 0 to 7.9 ± 1.2 (Group I) and 5.9 ± 1.3 µM AMC/mg protein (Group R) by day 4 (Fig. 5b). By day 7, the calpain activity further decreased to 6.3 ± 1.7 (Group I), 5.7 ± 0.9 (Group R) and 5.8 ± 1.1 µM AMC/mg protein (Group S) (*p* < 0.001). Collagenase activity showed no significant differences (*p* = 0.506) and declined from 2.1 ± 0.1 µM AMC/mg protein on day 0 to 1.5–1.6 µM AMC/mg protein on day 7 (Fig. 5c).

A similar decrease was observed in calpain-like activity in cod^41,42^ and super-chilled (surface-impregnated) salmon during ice storage^37^. There was a significantly higher calpain activity in the ice-stored I group than in the RSW-stored R group (*p* < 0.001). Gaarder, et al.^37^ reported a significantly higher calpain activity of super-chilled salmon at the beginning of ice storage, which the authors attributed to a higher calcium level in the cell cytosol than in ice-stored salmon. This presumably caused intracellular ice crystal formation during super-chilling (−25 °C), promoting protein denaturation and cell membrane disruption in super-chilled salmon. The lower calpain activity in the R group, as compared to the I group in this study, was not as expected, considering the higher drip loss and lower WHC, indicating accelerated denaturation in these samples in addition to the potential change in raw material properties during RSW storage (e.g. pH and solute concentration)^27,28^. The collagenase activity decreased first on day 7, later than the calpain activity, in agreement with the decrease in cod in correlation with the muscle softening during chilled storage^41,42^. However, there was little difference in the time course development of the calpain or collagenase activities that could account for the treatment-dependent effect on drip loss and WHC. RSW may alter solute concentrations (i.e. Ca^2+^, NaCl) and water distribution in fish muscle because of water- and salt uptake, which can enhance or hinder certain enzymes^37^. The responses of the different enzymes will further depend on subsequent storage conditions such as temperature and time^28,37,41^ where the use of modified atmosphere packaging is expected to play a role.

### Off-odour and microbiology

**Fig. 6:**
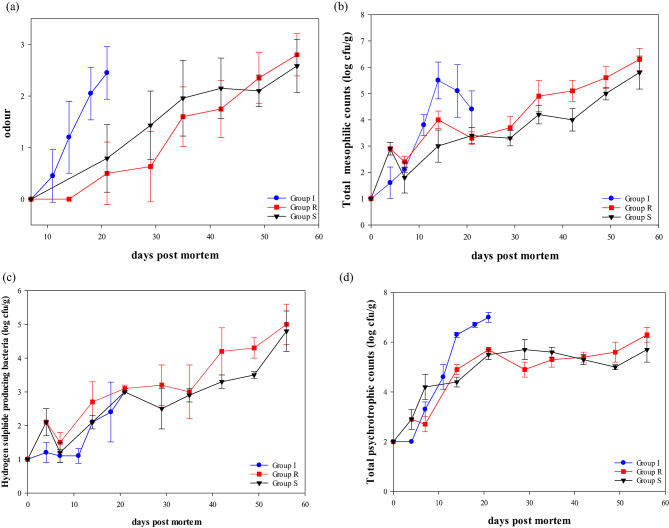
Graphs showing (**a**) off-odour after packaging (GLM; days: *p* < 0.001, group: *p* < 0.001, judge: *p* < 0.001). (**b**) Total mesophilic counts of whole fish (GLM; day: *p* < 0.001, group: *p* = 0.004) and portions after packaging (GLM; days: *p* < 0.001, group: *p* < 0.001). (**c**) Hydrogen sulphide-producing bacterial counts of whole fish (GLM; day: *p* < 0.001, group: *p* = 0.003) and portions after packaging (GLM; days: *p* < 0.001, group: *p* < 0.001). (**d**) Total psychrotrophic counts of whole fish (GLM; day: *p* < 0.001, group: *p* < 0.001) and portions after packaging (GLM; days: *p* < 0.001, group: *p* < 0.001).

Sensory off-odour analysis indicated that off-odour increased with time (Fig. 6a, *p* < 0.001). The control group was already spoiled after 18 days, while groups R and S had a better sensory shelf life of up to 49 days (*p* < 0.001). The off-odour analysis correlated to microbiology shelf life (TMC: *r* = 0.570, HSPB: *r* = 0.510, TPC: *r* = 0.437) and was negatively affected by the presence of oxygen in the headspace (*r*=−0.708). In addition, the judges significantly influenced the off-odour analyses (*p* < 0.001).

Figure 6b and d show the TMC, HSPB and TPC microbial growth throughout the experiment. The initial values were under the quantification limits, indicating that the raw material was of good microbial quality. Microbial growth increased through whole fish (TMC: *p* < 0.001, HSPB: *p* < 0.001, TPC: *p* < 0.001) and packaged storage (TMC: *p* < 0.001, HSPB: *p* < 0.001, TPC: *p* < 0.001). As an aerobic plate count of > 6 log cfu/g is considered the maximum acceptance^43^ the iced fish were spoiled after 14 days. This was in line with the results of Chan, et al.^29^ where fish initially stored on ice and then packaged in vacuum had a microbiological shelf life of 14 days. In contrast, groups R and S had a significantly better shelf life of around 49 days (TMC: *p* < 0.001, HSPB: *p* = 0.003, TPC: *p* < 0.001). This study presented a lower TMC and HSPB count for fish stored in RSW for 7 days compared to 4 days. Similar to previous studies^29,40,44^ RSW storage for whole fish gives a lower HSPB count. In addition, microbial growth was also higher for ice-stored salmon than those in RSW due to the greater availability of aerobic conditions^7^. Specific spoilage organisms (SSO) like HSPB produced by *Shewanella* spp. are present in fish and are responsible for the off-odours. In the present study, longer storage in RSW at −1 °C seemed to result in lower HSPB growth. This was, however, not prominent in the sensory analysis. The reason for the lower HSPB is not fully known. The low storage temperature and salt in RSW may act as a preservative and contribute to microbial inhibition.

Sivertsvik, et al.^31^ reported a synergistic effect on TPC between super-chilled storage at −2 °C and MAP of salmon fillets, resulting in a significantly better shelf life compared to chilled storage at 4 °C. Furthermore, Hansen, et al.^45^ demonstrated that super-chilling salmon fillets using a freezer tunnel combined with MAP and CO_2_ emitters prolonged the shelf life. However, they observed a higher drip loss and softer texture. For other species like Atlantic cod, super-chilling in slurry ice at −1.7 °C after MAP has also proven beneficial, providing better sensory and longer microbiological shelf life of > 32 days^46^. A recent study of Gurusamy, et al.^47^ presented the possibility of combining soluble gas stabilization and super-chilling to produce high quality salmon fillets. Pinto de Rezende, et al.^48^ summarized recent studies on super-chilling seafood products, with a consensus on extended shelf life, aligning with this study. Therefore, this study suggests that maintaining salmon in sub-chilled conditions, combined with MAP, can prolong the shelf life and remove the use of ice in the value chain. Nevertheless, proper temperature control is important. Cui, et al.^49^ reported that fluctuating temperatures of ± 2 °C with a targeted temperature of −3.5 °C during super-chilled storage can negatively influence the quality of salmon.

**Culture-independent microbial diversity**

**Fig. 7:**
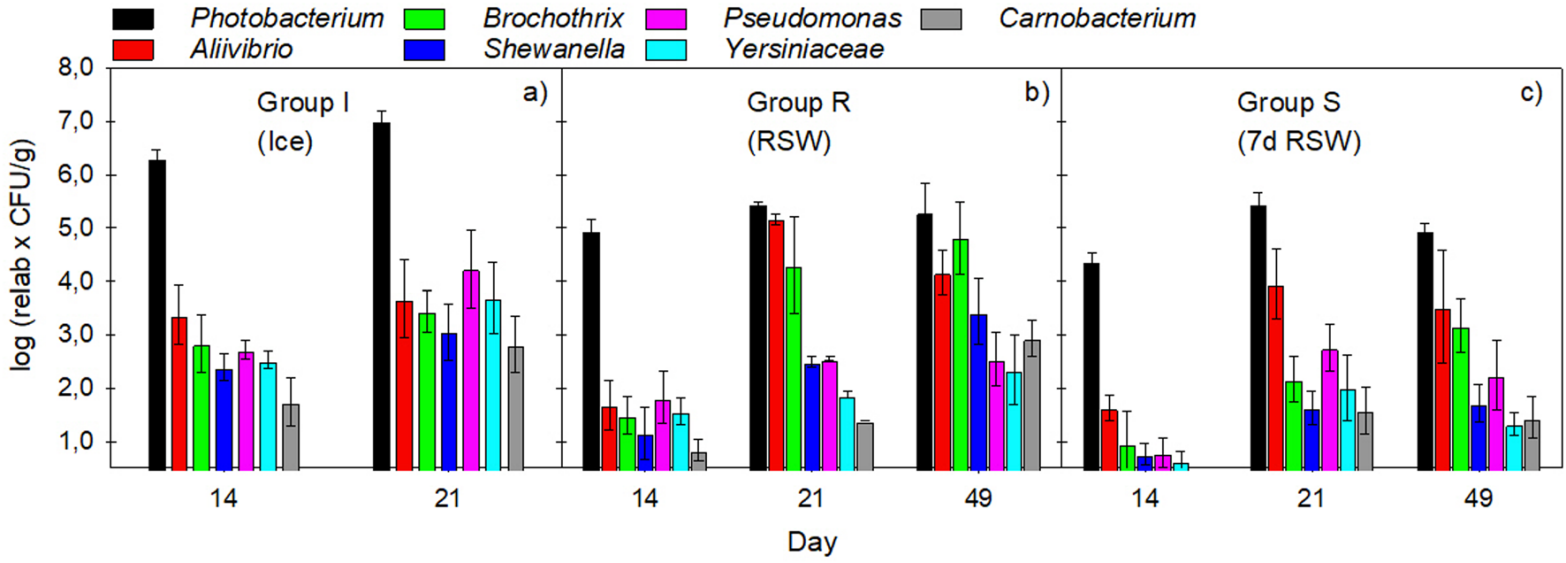
Estimated bacterial counts of the dominating taxa of (**a**) Group I, (**b**) Group R, and (**c**) Group S. The values are calculated based on the relative amounts (%) from gene sequencing and the total aerobic psychrotrophic counts of the samples (log_10_(relative values × CFU/g)).

The microbiota was dominated by *Photobacterium* independent of treatment (Fig. 7). On day 14, the relative abundance *of Photobacterium* in group S was 99.6%, which was like the two other groups (99.6 (R) and 99.7% (S)). After 21 days of storage, the relative abundance of *Photobacterium* declined to 70.7% in group R due to a relative increase in *Aliivibrio* (24.9%) and *Brochothrix* (3.8%) but was more stable in group I (98.6%) and group S (92.3%). In group S, the relative decline in *Photobacterium* was due to the growth of *Aliivibrio*, making up 7.3% of the total microbiota, with no increase in *Brochothrix* or other bacteria. After 49 days of storage, the microbiota of fish in group R had shifted to 68.2% *Photobacterium*, 24.0% *Brochothrix* and 6.2% *Aliivibrio*, compared to 85.5% *Photobacterium*, 2.0% *Brochothrix* and 12.0% *Aliivibrio* in group S. *Photobacterium* is quite resistant to CO_2_^50,51^ and its dominance in MAP fish is well-known^51,52^. Figure 7 shows that although *Photobacterium* dominates and is present in the region of approx. log 5 CFU/g after storage 21 and 49 days in both group R and group S, the levels of *Aliivibrio*,* Brochothrix* and *Shewanella* are significantly lower in group S compared to R at day 21 (*p* ≤ 0.021), and *Brochothrix* and *Shewanella* are significantly lower also at day 49 (*p* ≤ 0.007). *Shewanella and Aliivibrio* are also relatively psychrotolerant and resistant to CO_2_. Like *Photobacterium*, they give rise to off-odours through the production of trimethylamine (TMA), and together with *Brochothrix*, all are considered potent spoilers of MAP fish^53^. The present study indicates that prolonged storage in RSW may reduce the growth of these species of spoilage bacteria.

## Conclusion

This study presents the possibility of storing whole gutted salmon in RSW for 7 days and providing a good quality product with subsequent sub-chilled storage at −1 °C, with a substantial weight increase, better water holding capacity, and a higher water and salt content. Storing at −1 °C after MA packaging also resulted in lower CO_2_ levels than at 4 °C. In addition, our results showed that if strict temperature control is implemented, sub-chilling is a promising technology that can significantly extend the sensory and microbiological shelf life of fish and remove the use of ice after processing and packaging. This allows more space for extra fish to be transported over longer distances while maintaining a constant low temperature.

## Data Availability

The datasets used and/or analysed during the current study are available from the corresponding author on reasonable request.
